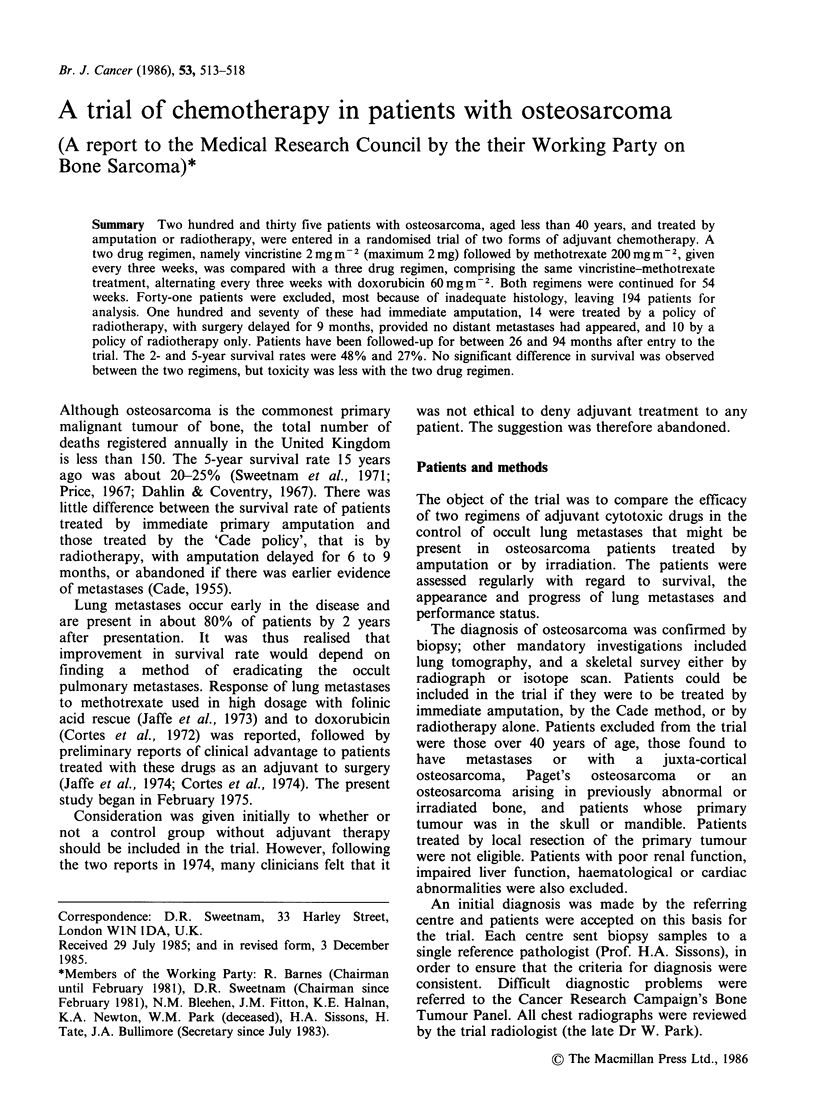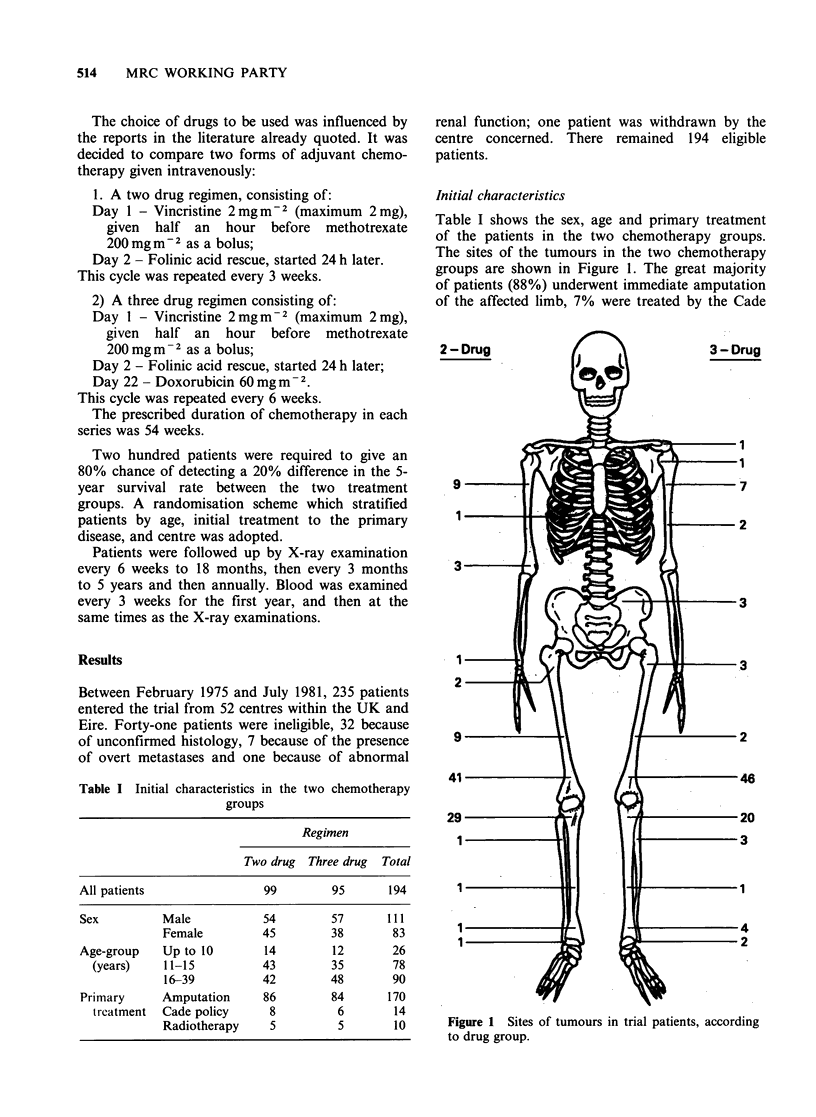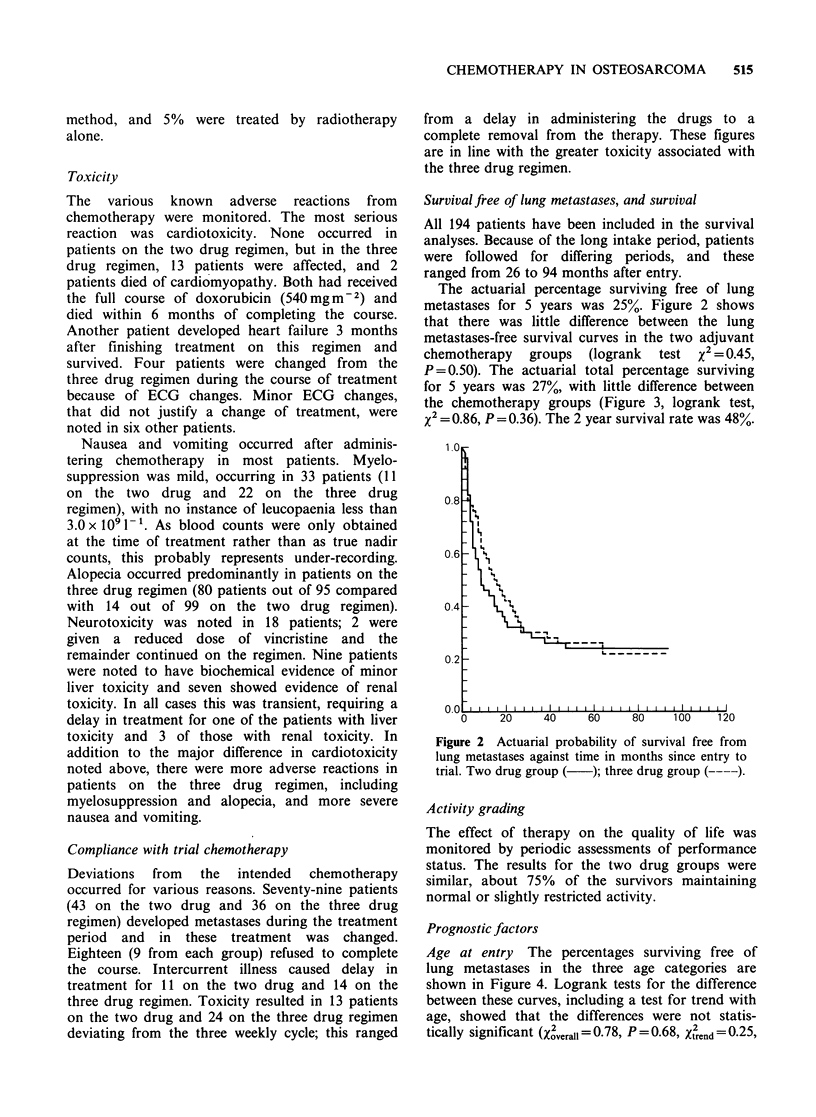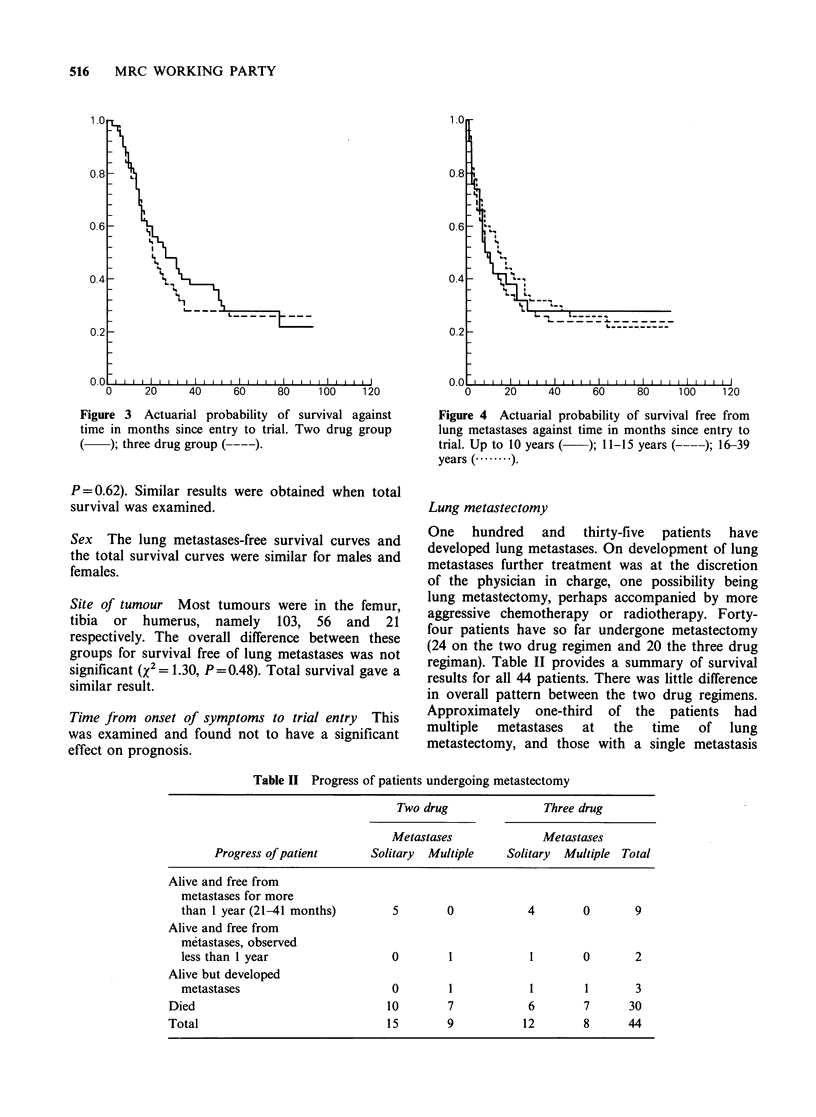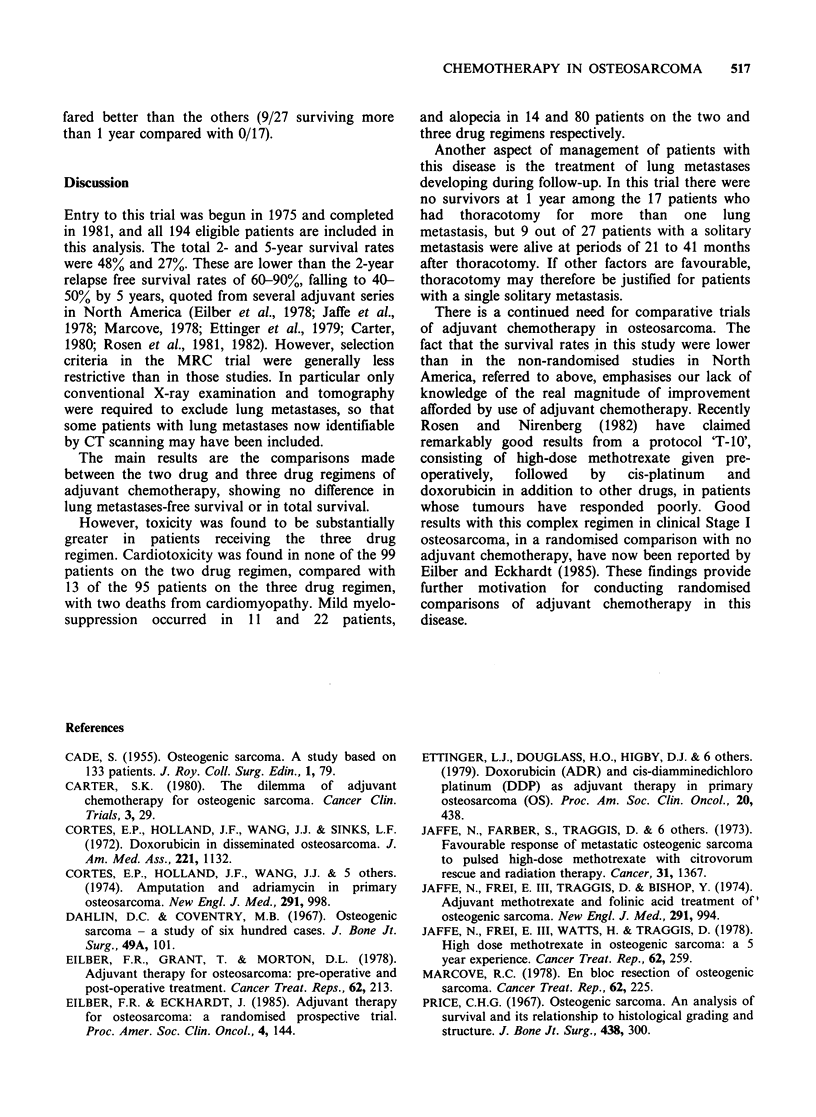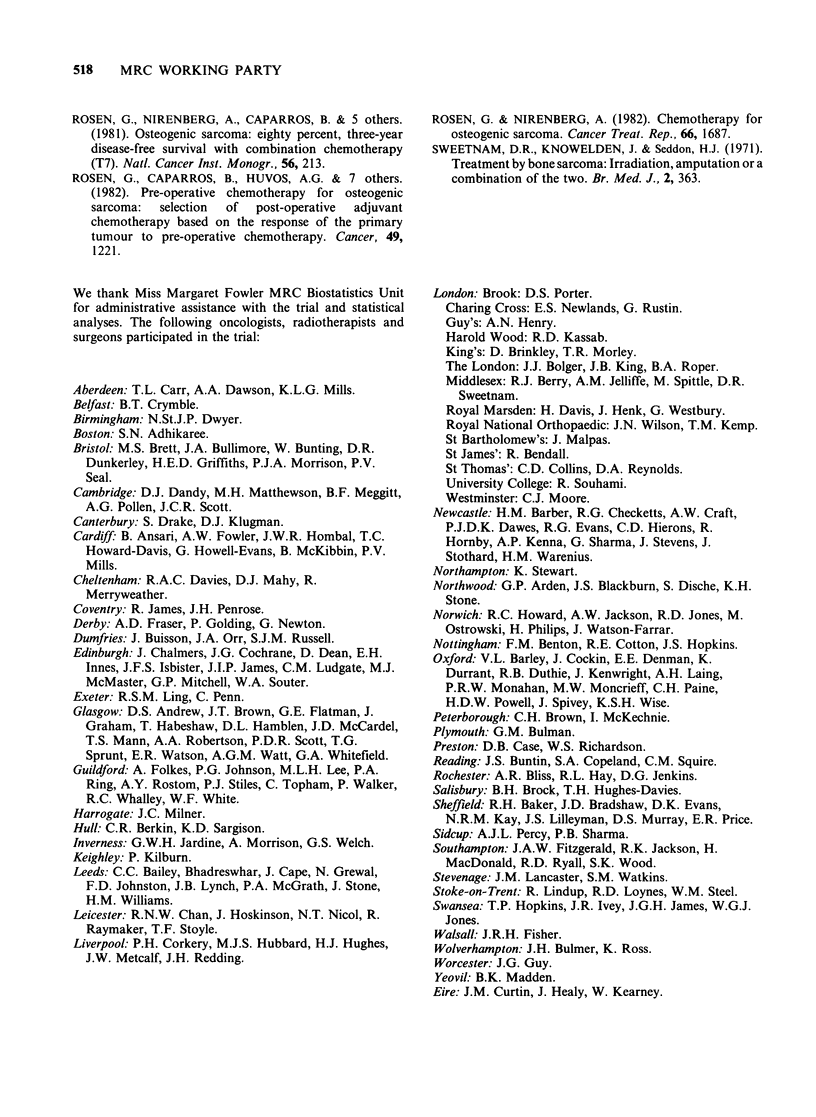# A trial of chemotherapy in patients with osteosarcoma (a report to the Medical Research Council by their Working Party on Bone Sarcoma.

**DOI:** 10.1038/bjc.1986.81

**Published:** 1986-04

**Authors:** 

## Abstract

Two hundred and thirty five patients with osteosarcoma, aged less than 40 years, and treated by amputation or radiotherapy, were entered in a randomised trial of two forms of adjuvant chemotherapy. A two drug regimen, namely vincristine 2 mg m-2 (maximum 2mg) followed by methotrexate 200 mgm-2, given every three weeks, was compared with a three drug regimen, comprising the same vincristine-methotrexate treatment, alternating every three weeks with doxorubicin 60 mg m-2. Both regimens were continued for 54 weeks. Forty-one patients were excluded, most because of inadequate histology, leaving 194 patients for analysis. One hundred and seventy of these had immediate amputation, 14 were treated by a policy of radiotherapy, with surgery delayed for 9 months, provided no distant metastases had appeared, and 10 by a policy of radiotherapy only. Patients have been followed-up for between 26 and 94 months after entry to the trial. The 2- and 5-year survival rates were 48% and 27%. No significant difference in survival was observed between the two regimens, but toxicity was less with the two drug regimen.


Br. J. Cancer (1986), **53**, 513–518

### A trial of chemotherapy in patients with osteosarcoma

### (A report to the Medical Research Council by the their Working Party on Bone Sarcoma)*

**Summary** Two hundred and thirty five patients with osteosarcoma, aged less than 40 years, and treated by amputation or radiotherapy, were entered in a randomised trial of two forms of adjuvant chemotherapy. A two drug regimen, namely vincristine 2 mg m$^{-2}$ (maximum 2 mg) followed by methotrexate 200 mg m$^{-2}$, given every three weeks, was compared with a three drug regimen, comprising the same vincristine–methotrexate treatment, alternating every three weeks with doxorubicin 60 mg m$^{-2}$. Both regimens were continued for 54 weeks. Forty-one patients were excluded, most because of inadequate histology, leaving 194 patients for analysis. One hundred and seventy of these had immediate amputation, 14 were treated by a policy of radiotherapy, with surgery delayed for 9 months, provided no distant metastases had appeared, and 10 by a policy of radiotherapy only. Patients have been followed-up for between 26 and 94 months after entry to the trial. The 2- and 5-year survival rates were 48% and 27%. No significant difference in survival was observed between the two regimens, but toxicity was less with the two drug regimen.

Although osteosarcoma is the commonest primary malignant tumour of bone, the total number of deaths registered annually in the United Kingdom is less than 150. The 5-year survival rate 15 years ago was about 20–25% (Sweetnam *et al.,* 1971; Price, 1967; Dahlin & Coventry, 1967). There was little difference between the survival rate of patients treated by immediate primary amputation and those treated by the 'Cade policy', that is by radiotherapy, with amputation delayed for 6 to 9 months, or abandoned if there was earlier evidence of metastases (Cade, 1955).

Lung metastases occur early in the disease and are present in about 80% of patients by 2 years after presentation. It was thus realised that improvement in survival rate would depend on finding a method of eradicating the occult pulmonary metastases. Response of lung metastases to methotrexate used in high dosage with folinic acid rescue (Jaffe *et al.,* 1973) and to doxorubicin (Cortes *et al.,* 1972) was reported, followed by preliminary reports of clinical advantage to patients treated with these drugs as an adjuvant to surgery (Jaffe *et al.,* 1974; Cortes *et al.,* 1974). The present study began in February 1975.

Consideration was given initially to whether or not a control group without adjuvant therapy should be included in the trial. However, following the two reports in 1974, many clinicians felt that it was not ethical to deny adjuvant treatment to any patient. The suggestion was therefore abandoned.

Correspondence: D.R. Sweetnam, 33 Harley Street, London W1N 1DA, U.K.
Received 29 July 1985; and in revised form, 3 December 1985.
*Members of the Working Party: R. Barnes (Chairman until February 1981), D.R. Sweetnam (Chairman since February 1981), N.M. Bleehen, J.M. Fitton, K.E. Halnan, K.A. Newton, W.M. Park (deceased), H.A. Sissons, H. Tate, J.A. Bullimore (Secretary since July 1983).

### Patients and methods

The object of the trial was to compare the efficacy of two regimens of adjuvant cytotoxic drugs in the control of occult lung metastases that might be present in osteosarcoma patients treated by amputation or by irradiation. The patients were assessed regularly with regard to survival, the appearance and progress of lung metastases and performance status.

The diagnosis of osteosarcoma was confirmed by biopsy; other mandatory investigations included lung tomography, and a skeletal survey either by radiograph or isotope scan. Patients could be included in the trial if they were to be treated by immediate amputation, by the Cade method, or by radiotherapy alone. Patients excluded from the trial were those over 40 years of age, those found to have metastases or with a juxta-cortical osteosarcoma, Paget's osteosarcoma or an osteosarcoma arising in previously abnormal or irradiated bone, and patients whose primary tumour was in the skull or mandible. Patients treated by local resection of the primary tumour were not eligible. Patients with poor renal function, impaired liver function, haematological or cardiac abnormalities were also excluded.

An initial diagnosis was made by the referring centre and patients were accepted on this basis for the trial. Each centre sent biopsy samples to a single reference pathologist (Prof. H.A. Sissons), in order to ensure that the criteria for diagnosis were consistent. Difficult diagnostic problems were referred to the Cancer Research Campaign's Bone Tumour Panel. All chest radiographs were reviewed by the trial radiologist (the late Dr W. Park).

© The Macmillan Press Ltd., 1986

The choice of drugs to be used was influenced by the reports in the literature already quoted. It was decided to compare two forms of adjuvant chemotherapy given intravenously:

1. A two drug regimen, consisting of:
Day 1 – Vincristine $2\,mg\,m^{-2}$ (maximum 2 mg), given half an hour before methotrexate $200\,mg\,m^{-2}$ as a bolus;
Day 2 – Folinic acid rescue, started 24 h later.
This cycle was repeated every 3 weeks.

2) A three drug regimen consisting of:
Day 1 – Vincristine $2\,mg\,m^{-2}$ (maximum 2 mg), given half an hour before methotrexate $200\,mg\,m^{-2}$ as a bolus;
Day 2 – Folinic acid rescue, started 24 h later;
Day 22 – Doxorubicin $60\,mg\,m^{-2}$.
This cycle was repeated every 6 weeks.

The prescribed duration of chemotherapy in each series was 54 weeks.

Two hundred patients were required to give an 80% chance of detecting a 20% difference in the 5-year survival rate between the two treatment groups. A randomisation scheme which stratified patients by age, initial treatment to the primary disease, and centre was adopted.

Patients were followed up by X-ray examination every 6 weeks to 18 months, then every 3 months to 5 years and then annually. Blood was examined every 3 weeks for the first year, and then at the same times as the X-ray examinations.

### Results

Between February 1975 and July 1981, 235 patients entered the trial from 52 centres within the UK and Eire. Forty-one patients were ineligible, 32 because of unconfirmed histology, 7 because of the presence of overt metastases and one because of abnormal renal function; one patient was withdrawn by the centre concerned. There remained 194 eligible patients.

**Table I** Initial characteristics in the two chemotherapy groups

| | | *Regimen* | | |
|---|---|---|---|---|
| | | *Two drug* | *Three drug* | *Total* |
| All patients | | 99 | 95 | 194 |
| Sex | Male | 54 | 57 | 111 |
| | Female | 45 | 38 | 83 |
| Age-group (years) | Up to 10 | 14 | 12 | 26 |
| | 11–15 | 43 | 35 | 78 |
| | 16–39 | 42 | 48 | 90 |
| Primary treatment | Amputation | 86 | 84 | 170 |
| | Cade policy | 8 | 6 | 14 |
| | Radiotherapy | 5 | 5 | 10 |

### *Initial characteristics*

Table I shows the sex, age and primary treatment of the patients in the two chemotherapy groups. The sites of the tumours in the two chemotherapy groups are shown in Figure 1. The great majority of patients (88%) underwent immediate amputation of the affected limb, 7% were treated by the Cade

**Figure 1** Sites of tumours in trial patients, according to drug group.

method, and 5% were treated by radiotherapy alone.

*Toxicity*

The various known adverse reactions from chemotherapy were monitored. The most serious reaction was cardiotoxicity. None occurred in patients on the two drug regimen, but in the three drug regimen, 13 patients were affected, and 2 patients died of cardiomyopathy. Both had received the full course of doxorubicin ($540\,mg\,m^{-2}$) and died within 6 months of completing the course. Another patient developed heart failure 3 months after finishing treatment on this regimen and survived. Four patients were changed from the three drug regimen during the course of treatment because of ECG changes. Minor ECG changes, that did not justify a change of treatment, were noted in six other patients.

Nausea and vomiting occurred after administering chemotherapy in most patients. Myelosuppression was mild, occurring in 33 patients (11 on the two drug and 22 on the three drug regimen), with no instance of leucopaenia less than $3.0 \times 10^9\,l^{-1}$. As blood counts were only obtained at the time of treatment rather than as true nadir counts, this probably represents under-recording. Alopecia occurred predominantly in patients on the three drug regimen (80 patients out of 95 compared with 14 out of 99 on the two drug regimen). Neurotoxicity was noted in 18 patients; 2 were given a reduced dose of vincristine and the remainder continued on the regimen. Nine patients were noted to have biochemical evidence of minor liver toxicity and seven showed evidence of renal toxicity. In all cases this was transient, requiring a delay in treatment for one of the patients with liver toxicity and 3 of those with renal toxicity. In addition to the major difference in cardiotoxicity noted above, there were more adverse reactions in patients on the three drug regimen, including myelosuppression and alopecia, and more severe nausea and vomiting.

*Compliance with trial chemotherapy*

Deviations from the intended chemotherapy occurred for various reasons. Seventy-nine patients (43 on the two drug and 36 on the three drug regimen) developed metastases during the treatment period and in these treatment was changed. Eighteen (9 from each group) refused to complete the course. Intercurrent illness caused delay in treatment for 11 on the two drug and 14 on the three drug regimen. Toxicity resulted in 13 patients on the two drug and 24 on the three drug regimen deviating from the three weekly cycle; this ranged from a delay in administering the drugs to a complete removal from the therapy. These figures are in line with the greater toxicity associated with the three drug regimen.

*Survival free of lung metastases, and survival*

All 194 patients have been included in the survival analyses. Because of the long intake period, patients were followed for differing periods, and these ranged from 26 to 94 months after entry.

The actuarial percentage surviving free of lung metastases for 5 years was 25%. Figure 2 shows that there was little difference between the lung metastases-free survival curves in the two adjuvant chemotherapy groups (logrank test $\chi^2=0.45$, $P=0.50$). The actuarial total percentage surviving for 5 years was 27%, with little difference between the chemotherapy groups (Figure 3, logrank test, $\chi^2=0.86$, $P=0.36$). The 2 year survival rate was 48%.

**Figure 2** Actuarial probability of survival free from lung metastases against time in months since entry to trial. Two drug group (——); three drug group (----).

*Activity grading*

The effect of therapy on the quality of life was monitored by periodic assessments of performance status. The results for the two drug groups were similar, about 75% of the survivors maintaining normal or slightly restricted activity.

*Prognostic factors*

*Age at entry* The percentages surviving free of lung metastases in the three age categories are shown in Figure 4. Logrank tests for the difference between these curves, including a test for trend with age, showed that the differences were not statistically significant ($\chi^2_{overall}=0.78$, $P=0.68$, $\chi^2_{trend}=0.25$,

**Figure 3** Actuarial probability of survival against time in months since entry to trial. Two drug group (——); three drug group (----).

**Figure 4** Actuarial probability of survival free from lung metastases against time in months since entry to trial. Up to 10 years (——); 11–15 years (----); 16–39 years (········).

$P = 0.62$). Similar results were obtained when total survival was examined.

*Sex* The lung metastases-free survival curves and the total survival curves were similar for males and females.

*Site of tumour* Most tumours were in the femur, tibia or humerus, namely 103, 56 and 21 respectively. The overall difference between these groups for survival free of lung metastases was not significant ($\chi^2 = 1.30$, $P = 0.48$). Total survival gave a similar result.

*Time from onset of symptoms to trial entry* This was examined and found not to have a significant effect on prognosis.

*Lung metastectomy*

One hundred and thirty-five patients have developed lung metastases. On development of lung metastases further treatment was at the discretion of the physician in charge, one possibility being lung metastectomy, perhaps accompanied by more aggressive chemotherapy or radiotherapy. Forty-four patients have so far undergone metastectomy (24 on the two drug regimen and 20 the three drug regiman). Table II provides a summary of survival results for all 44 patients. There was little difference in overall pattern between the two drug regimens. Approximately one-third of the patients had multiple metastases at the time of lung metastectomy, and those with a single metastasis

**Table II** Progress of patients undergoing metastectomy

| | *Two drug* | | *Three drug* | | |
|---|---|---|---|---|---|
| | *Metastases* | | *Metastases* | | |
| *Progress of patient* | *Solitary* | *Multiple* | *Solitary* | *Multiple* | *Total* |
| Alive and free from metastases for more than 1 year (21–41 months) | 5 | 0 | 4 | 0 | 9 |
| Alive and free from mètastases, observed less than 1 year | 0 | 1 | 1 | 0 | 2 |
| Alive but developed metastases | 0 | 1 | 1 | 1 | 3 |
| Died | 10 | 7 | 6 | 7 | 30 |
| Total | 15 | 9 | 12 | 8 | 44 |

fared better than the others (9/27 surviving more than 1 year compared with 0/17).

### Discussion

Entry to this trial was begun in 1975 and completed in 1981, and all 194 eligible patients are included in this analysis. The total 2- and 5-year survival rates were 48% and 27%. These are lower than the 2-year relapse free survival rates of 60–90%, falling to 40–50% by 5 years, quoted from several adjuvant series in North America (Eilber *et al.*, 1978; Jaffe *et al.*, 1978; Marcove, 1978; Ettinger *et al.*, 1979; Carter, 1980; Rosen *et al.*, 1981, 1982). However, selection criteria in the MRC trial were generally less restrictive than in those studies. In particular only conventional X-ray examination and tomography were required to exclude lung metastases, so that some patients with lung metastases now identifiable by CT scanning may have been included.

The main results are the comparisons made between the two drug and three drug regimens of adjuvant chemotherapy, showing no difference in lung metastases-free survival or in total survival.

However, toxicity was found to be substantially greater in patients receiving the three drug regimen. Cardiotoxicity was found in none of the 99 patients on the two drug regimen, compared with 13 of the 95 patients on the three drug regimen, with two deaths from cardiomyopathy. Mild myelosuppression occurred in 11 and 22 patients, and alopecia in 14 and 80 patients on the two and three drug regimens respectively.

Another aspect of management of patients with this disease is the treatment of lung metastases developing during follow-up. In this trial there were no survivors at 1 year among the 17 patients who had thoracotomy for more than one lung metastasis, but 9 out of 27 patients with a solitary metastasis were alive at periods of 21 to 41 months after thoracotomy. If other factors are favourable, thoracotomy may therefore be justified for patients with a single solitary metastasis.

There is a continued need for comparative trials of adjuvant chemotherapy in osteosarcoma. The fact that the survival rates in this study were lower than in the non-randomised studies in North America, referred to above, emphasises our lack of knowledge of the real magnitude of improvement afforded by use of adjuvant chemotherapy. Recently Rosen and Nirenberg (1982) have claimed remarkably good results from a protocol 'T-10', consisting of high-dose methotrexate given pre-operatively, followed by cis-platinum and doxorubicin in addition to other drugs, in patients whose tumours have responded poorly. Good results with this complex regimen in clinical Stage I osteosarcoma, in a randomised comparison with no adjuvant chemotherapy, have now been reported by Eilber and Eckhardt (1985). These findings provide further motivation for conducting randomised comparisons of adjuvant chemotherapy in this disease.

### References

CADE, S. (1955). Osteogenic sarcoma. A study based on 133 patients. *J. Roy. Coll. Surg. Edin.*, **1,** 79.

CARTER, S.K. (1980). The dilemma of adjuvant chemotherapy for osteogenic sarcoma. *Cancer Clin. Trials,* **3,** 29.

CORTES, E.P., HOLLAND, J.F., WANG, J.J. & SINKS, L.F. (1972). Doxorubicin in disseminated osteosarcoma. *J. Am. Med. Ass.*, **221,** 1132.

CORTES, E.P., HOLLAND, J.F., WANG, J.J. & 5 others. (1974). Amputation and adriamycin in primary osteosarcoma. *New Engl. J. Med.*, **291,** 998.

DAHLIN, D.C. & COVENTRY, M.B. (1967). Osteogenic sarcoma – a study of six hundred cases. *J. Bone Jt. Surg.*, **49A,** 101.

EILBER, F.R., GRANT, T. & MORTON, D.L. (1978). Adjuvant therapy for osteosarcoma: pre-operative and post-operative treatment. *Cancer Treat. Reps.*, **62,** 213.

EILBER, F.R. & ECKHARDT, J. (1985). Adjuvant therapy for osteosarcoma: a randomised prospective trial. *Proc. Amer. Soc. Clin. Oncol.*, **4,** 144.

ETTINGER, L.J., DOUGLASS, H.O., HIGBY, D.J. & 6 others. (1979). Doxorubicin (ADR) and cis-diamminedichloro platinum (DDP) as adjuvant therapy in primary osteosarcoma (OS). *Proc. Am. Soc. Clin. Oncol.*, **20,** 438.

JAFFE, N., FARBER, S., TRAGGIS, D. & 6 others. (1973). Favourable response of metastatic osteogenic sarcoma to pulsed high-dose methotrexate with citrovorum rescue and radiation therapy. *Cancer,* **31,** 1367.

JAFFE, N., FREI, E. III, TRAGGIS, D. & BISHOP, Y. (1974). Adjuvant methotrexate and folinic acid treatment of osteogenic sarcoma. *New Engl. J. Med.*, **291,** 994.

JAFFE, N., FREI, E. III, WATTS, H. & TRAGGIS, D. (1978). High dose methotrexate in osteogenic sarcoma: a 5 year experience. *Cancer Treat. Rep.*, **62,** 259.

MARCOVE, R.C. (1978). En bloc resection of osteogenic sarcoma. *Cancer Treat. Rep.*, **62,** 225.

PRICE, C.H.G. (1967). Osteogenic sarcoma. An analysis of survival and its relationship to histological grading and structure. *J. Bone Jt. Surg.*, **438,** 300.

ROSEN, G., NIRENBERG, A., CAPARROS, B. & 5 others. (1981). Osteogenic sarcoma: eighty percent, three-year disease-free survival with combination chemotherapy (T7). *Natl. Cancer Inst. Monogr.*, **56**, 213.

ROSEN, G., CAPARROS, B., HUVOS, A.G. & 7 others. (1982). Pre-operative chemotherapy for osteogenic sarcoma: selection of post-operative adjuvant chemotherapy based on the response of the primary tumour to pre-operative chemotherapy. *Cancer*, **49**, 1221.

ROSEN, G. & NIRENBERG, A. (1982). Chemotherapy for osteogenic sarcoma. *Cancer Treat. Rep.*, **66**, 1687.

SWEETNAM, D.R., KNOWELDEN, J. & Seddon, H.J. (1971). Treatment by bone sarcoma: Irradiation, amputation or a combination of the two. *Br. Med. J.*, **2**, 363.

We thank Miss Margaret Fowler MRC Biostatistics Unit for administrative assistance with the trial and statistical analyses. The following oncologists, radiotherapists and surgeons participated in the trial:

*Aberdeen:* T.L. Carr, A.A. Dawson, K.L.G. Mills.
*Belfast:* B.T. Crymble.
*Birmingham:* N.St.J.P. Dwyer.
*Boston:* S.N. Adhikaree.
*Bristol:* M.S. Brett, J.A. Bullimore, W. Bunting, D.R. Dunkerley, H.E.D. Griffiths, P.J.A. Morrison, P.V. Seal.
*Cambridge:* D.J. Dandy, M.H. Matthewson, B.F. Meggitt, A.G. Pollen, J.C.R. Scott.
*Canterbury:* S. Drake, D.J. Klugman.
*Cardiff:* B. Ansari, A.W. Fowler, J.W.R. Hombal, T.C. Howard-Davis, G. Howell-Evans, B. McKibbin, P.V. Mills.
*Cheltenham:* R.A.C. Davies, D.J. Mahy, R. Merryweather.
*Coventry:* R. James, J.H. Penrose.
*Derby:* A.D. Fraser, P. Golding, G. Newton.
*Dumfries:* J. Buisson, J.A. Orr, S.J.M. Russell.
*Edinburgh:* J. Chalmers, J.G. Cochrane, D. Dean, E.H. Innes, J.F.S. Isbister, J.I.P. James, C.M. Ludgate, M.J. McMaster, G.P. Mitchell, W.A. Souter.
*Exeter:* R.S.M. Ling, C. Penn.
*Glasgow:* D.S. Andrew, J.T. Brown, G.E. Flatman, J. Graham, T. Habeshaw, D.L. Hamblen, J.D. McCardel, T.S. Mann, A.A. Robertson, P.D.R. Scott, T.G. Sprunt, E.R. Watson, A.G.M. Watt, G.A. Whitefield.
*Guildford:* A. Folkes, P.G. Johnson, M.L.H. Lee, P.A. Ring, A.Y. Rostom, P.J. Stiles, C. Topham, P. Walker, R.C. Whalley, W.F. White.
*Harrogate:* J.C. Milner.
*Hull:* C.R. Berkin, K.D. Sargison.
*Inverness:* G.W.H. Jardine, A. Morrison, G.S. Welch.
*Keighley:* P. Kilburn.
*Leeds:* C.C. Bailey, Bhadreswhar, J. Cape, N. Grewal, F.D. Johnston, J.B. Lynch, P.A. McGrath, J. Stone, H.M. Williams.
*Leicester:* R.N.W. Chan, J. Hoskinson, N.T. Nicol, R. Raymaker, T.F. Stoyle.
*Liverpool:* P.H. Corkery, M.J.S. Hubbard, H.J. Hughes, J.W. Metcalf, J.H. Redding.
*London:* Brook: D.S. Porter.
Charing Cross: E.S. Newlands, G. Rustin.
Guy's: A.N. Henry.
Harold Wood: R.D. Kassab.
King's: D. Brinkley, T.R. Morley.
The London: J.J. Bolger, J.B. King, B.A. Roper.
Middlesex: R.J. Berry, A.M. Jelliffe, M. Spittle, D.R. Sweetnam.
Royal Marsden: H. Davis, J. Henk, G. Westbury.
Royal National Orthopaedic: J.N. Wilson, T.M. Kemp.
St Bartholomew's: J. Malpas.
St James': R. Bendall.
St Thomas': C.D. Collins, D.A. Reynolds.
University College: R. Souhami.
Westminster: C.J. Moore.
*Newcastle:* H.M. Barber, R.G. Checketts, A.W. Craft, P.J.D.K. Dawes, R.G. Evans, C.D. Hierons, R. Hornby, A.P. Kenna, G. Sharma, J. Stevens, J. Stothard, H.M. Warenius.
*Northampton:* K. Stewart.
*Northwood:* G.P. Arden, J.S. Blackburn, S. Dische, K.H. Stone.
*Norwich:* R.C. Howard, A.W. Jackson, R.D. Jones, M. Ostrowski, H. Philips, J. Watson-Farrar.
*Nottingham:* F.M. Benton, R.E. Cotton, J.S. Hopkins.
*Oxford:* V.L. Barley, J. Cockin, E.E. Denman, K. Durrant, R.B. Duthie, J. Kenwright, A.H. Laing, P.R.W. Monahan, M.W. Moncrieff, C.H. Paine, H.D.W. Powell, J. Spivey, K.S.H. Wise.
*Peterborough:* C.H. Brown, I. McKechnie.
*Plymouth:* G.M. Bulman.
*Preston:* D.B. Case, W.S. Richardson.
*Reading:* J.S. Buntin, S.A. Copeland, C.M. Squire.
*Rochester:* A.R. Bliss, R.L. Hay, D.G. Jenkins.
*Salisbury:* B.H. Brock, T.H. Hughes-Davies.
*Sheffield:* R.H. Baker, J.D. Bradshaw, D.K. Evans, N.R.M. Kay, J.S. Lilleyman, D.S. Murray, E.R. Price.
*Sidcup:* A.J.L. Percy, P.B. Sharma.
*Southampton:* J.A.W. Fitzgerald, R.K. Jackson, H. MacDonald, R.D. Ryall, S.K. Wood.
*Stevenage:* J.M. Lancaster, S.M. Watkins.
*Stoke-on-Trent:* R. Lindup, R.D. Loynes, W.M. Steel.
*Swansea:* T.P. Hopkins, J.R. Ivey, J.G.H. James, W.G.J. Jones.
*Walsall:* J.R.H. Fisher.
*Wolverhampton:* J.H. Bulmer, K. Ross.
*Worcester:* J.G. Guy.
*Yeovil:* B.K. Madden.
*Eire:* J.M. Curtin, J. Healy, W. Kearney.